# Plant Development Shapes the Rhizosphere Microbiota Assembly of *Cedrela odorata* (Meliaceae)

**DOI:** 10.3390/microorganisms14050997

**Published:** 2026-04-29

**Authors:** Carlos Cadena-Lozano, Jesús Alejandro Zamora-Briseño, Ioreni Margarita Hernández-Velázquez, Laura Yesenia Solís-Ramos, Alejandro Antonio Castro Luna, Alejandro Pereira-Santana, Antonio Andrade-Torres

**Affiliations:** 1 Biotecnología y Ecología de Organismos Simbióticos, CAUV-173 Ecología y Manejo de la Biodiversidad, INBIOTECA (Instituto de Biotecnología y Ecología Aplicada), Universidad Veracruzana, Av. de las Culturas Veracruzanas No. 101, Col. E. Zapata, Xalapa 91090, Veracruz, Mexico; zs23000363@estudiantes.uv.mx (C.C.-L.); alcastro@uv.mx (A.A.C.L.); 2 Red de Estudios Moleculares Avanzados, Campus III, Instituto de Ecología A. C., Carretera Antigua a Coatepec 351, Xalapa 91073, Veracruz, Mexico; alejandro.zamora@inecol.mx (J.A.Z.-B.); ihernandez@inecol.mx (I.M.H.-V.); 3 Biotecnología de Plantas y Hongos Micorrícicos Arbusculares (Biotec-PYHMA), Escuela de Biología y Centro de Investigación en Biodiversidad y Ecología Tropical (CIBET), Universidad de Costa Rica, San Pedro de Montes de Oca, San José 11501-2060, Costa Rica; laura.solisramos@ucr.ac.cr; 4 SECIHTI-Centro de Investigación y Asistencia en Tecnología y Diseño del Estado de Jalisco, Sede Sureste, Tablaje Catastral 31264 Km. 5.5 Carr. Sierra Papacal-Chuburna Pto., Parque Científico Tecnológico de Yucatán, Mérida 97302, Yucatán, Mexico

**Keywords:** microbiota, microbial communities, metabarcoding

## Abstract

*Cedrela odorata* L. (Meliaceae), commonly known as Spanish cedar, is a timber species of high interest for mass propagation. However, there are factors that complicate this process, such as poor natural regeneration, rapid loss of seed viability, or slow growth. In this context, examining the rhizosphere microbiota of this species may help devise strategies to improve its establishment during early development. Thus, we conducted a meta-taxonomic analysis of the bacterial and fungal communities associated with the rhizosphere of seedlings and adults of *C. odorata* and the surrounding bulk soil. We found that the alpha diversity of the microbiota in the rhizosphere was not significantly different between adults, seedlings, and soil samples, whereas the beta diversity showed significant differences between soil and rhizosphere and between developmental stages. We identified several differential genera of bacteria and fungi, including nitrogen-fixing bacteria such as *Bradyrhizobium* and *Pseudolabrys*, that could play a beneficial role in the establishment and development of Spanish cedar. This is the first study that surveyed the microbiota associated with Spanish cedar, and the findings obtained may help guide further functional studies and to develop knowledge-based microbial inocula to improve the establishment of this species under field conditions.

## 1. Introduction

Spanish cedar, *Cedrela odorata* L. (Meliaceae), is a timber species that can reach up to 45 m in height and is found in areas with temperatures between 11 and 38 °C and annual rainfall of 1200 to 2000 mm [1]. It is biogeographically distributed from northern Mexico to northern Argentina, where it is a natural component of tropical rainforests [1]. This species is of interest because of its high-quality wood, but its commercial propagation is difficult due to issues with natural regeneration, rapid loss of seed viability, slow growth, and damage caused by the shoot borer, *Hypsipyla grandella* Zeller (Lepidoptera: Pyralidae) [1,2,3]. Spanish cedar is included in the lists of international organizations such as IUCN and CITES, and protected by NOM-059 in Mexico [1].

The rhizosphere is the zone of soil influenced by root systems, where roots, mucigel, microorganisms, macroorganisms, and soil structure and nutrients interact dynamically [4,5]. During these interactions, the microorganisms inhabiting the rhizosphere (e.g., rhizosphere microbiota) become involved in several functions, such as plant growth and nutrition, and provide resistance to abiotic stress factors and against pathogens [6,7]. The rhizosphere microbiota can be critically important to plants, acting as a major driver of growth and establishment [8]. Within the rhizosphere microbiota, there is a wide array of beneficial microorganisms that have been extensively studied for their relevance in the establishment, growth, and development of plants, such as fungal mycorrhizae, growth-promoting rhizobacteria, or pathogen-suppressive microorganisms [9,10].

Plants perform a functional selection of soil microorganisms in the rhizosphere, and thus the rhizosphere is often less diverse than the bulk soil [11]. The assembly of the rhizosphere microbiota is dependent on intrinsic plant factors (e.g., species, genotype, tissue type, chemical signaling, exudates, immunity, stage of development), microbial interactions, and environmental factors such as soil characteristics, cultivation practices, and climatic conditions [12,13,14,15,16,17]. Thus, given the symbiotic relationship between plants and their microbiota, it is evident that the latter can also be affected by the metabolic conditions of the former. There is strong evidence that plants have a differential influence on their accompanying microorganisms at different developmental stages, changing their function and composition through deterministic selection during growth [18,19]. Some plants are capable of secreting protective compounds in their roots, which provide defense against certain bacteria during mature life stages [18,20,21]. Metabolic changes caused by growth can influence the structure of the rhizosphere microbiota, while compounds produced by rhizobacteria, such as plant growth regulators, can play important roles in mitigating abiotic stresses in the host plants [22,23]. Therefore, understanding the effects of plant growth stage changes on the microbiota opens promising avenues for rhizosphere manipulation and development for sustainable crop production [8].

In plant nurseries, forest soil is commonly used as a substrate for propagated plants, but this practice can cause ecological damage because of excessive soil removal. By doing so, farmers or foresters also force plants to adapt to foreign microbiota, often overlooking the importance of the long-lasting, evolutionarily conserved symbiotic interactions established between plants and microorganisms [24,25,26,27]. Furthermore, one common agricultural practice is to use sterile soil or other substrates to prevent the spread of pathogens, which can lead to the loss of important microorganisms that are necessary for plant growth [28]. This practice is counterintuitive when we consider numerous studies showing that symbiotic microorganisms are essential for the successful establishment of forest plantations [29], including nursery experiments demonstrating that mycorrhizal fungi and rhizobacteria enhance the development of Spanish cedar [30,31,32]. More than 50 taxa of arbuscular mycorrhizal fungi have been identified in the rhizosphere of *C. odorata*, although the microbial community it may harbor is still unknown [33,34,35]. Under this scenario, the use of non-native microorganisms is discouraged as it leads to the introduction of foreign microorganisms into the ecosystem, potentially causing major ecological problems [28,36,37]. In this sense, the identification of native plant-associated microorganisms can aid in the customized design of microbial consortia for the fertilization of commercially propagated plants.

Based on the above, in the present study, we hypothesized that *C. odorata* recruits a defined microbiota that changes according to the developmental stage of the plants. Testing this hypothesis enabled the identification of microbial candidates for the development of formulations with the potential to improve the establishment of Spanish cedar in nurseries.

## 2. Materials and Methods

### 2.1. Sampling

We sampled a Spanish cedar orchard in the municipality of Tlaltetela, in the central mountainous region of the state of Veracruz, Mexico (19°19′ N and 96°54′ W), at 960 m above sea level. The orchard is located in the central part of the state, on the foothills of the Sierra Madre Oriental, and the surrounding landscape is composed of temperate deciduous forests. The climate is temperate-humid, with an average annual temperature of 18 °C and average annual rainfall of 1800 mm. The sampling was carried out in December 2022, during the dry season.

We collected soil rhizosphere samples from six adult *C. odorata* trees separated by at least 15 m (i.e., diameter greater than 30 cm, with evidence of having produced seeds and a healthy appearance) and 6 seedlings separated by at least 5 m (individuals of less than 30 cm in height with a healthy appearance). The rhizosphere samples were collected from the four cardinal points around the trunk of each tree at a depth of 4–10 cm by removing the organic matter and carefully extracting the samples. We took composite samples of fine roots measuring ~30 cm in length. After shaking and scraping the soil particles off the roots with a sterile microspatula, the rhizosphere was placed in sterile 50 mL plastic tubes for storage. We also included six bulk soil samples that were not influenced by the roots of the Spanish cedars (collected at a distance of at least 10 m from the trees) and pooled them into one compound sample. A compound Soil subsample was sent to FertiLab, Celaya, Guanajuato, Mexico, for the determination of moisture percentage, electrical conductivity, potassium, calcium, magnesium, iron, zinc, manganese, sodium, copper, and available phosphorus concentrations, as well as bulk density, field capacity, organic carbon, organic matter, permanent wilting point, pH, total nitrogen (NO_3_^−^, NH_4_^+^), texture, and cation exchange capacity.

### 2.2. DNA Extraction, Library Construction, and Sequencing

DNA extraction was carried out with the DNeasy PowerSoil Pro Kit (Qiagen, Hilden, Germany) using 200 mg of each sample, accounting for a total of six replicates per condition. The concentration and quality of the DNA in each sample were determined spectrophotometrically using a NanoDrop 2000 spectrophotometer (Thermo Scientific, Wilmington, DE, USA), and DNA integrity was assessed using a 1% agarose gel.

Targeted sequencing of the 16S rRNA gene for prokaryote identification and ITS of the 5.8S gene for fungal identification was performed by Novogene Co. (Sacramento, CA, USA). The V3-V4 region of the bacterial 16S rRNA gene was amplified from gDNA derived from the samples using the forward (341F 5′-CCT AYG GGR BGC ASC AG-3′) and the reverse (806R 5′-GGA CTA CNN GGG TAT CTA AT-3′) primers (Novogene Co., Sacramento, CA, USA), while the ITS was amplified using the forward (ITS5-1737F 5′-G GAA GTA AAA GTC GTA ACA AGG) and the reverse (ITS2-2043R 5′-GC TGC GTT CTT CAT CGA TGC) primers for the ITS1 region. All PCR reactions were carried out with Phusion^®^ High-Fidelity PCR Master Mix (New England Biolabs, Ipswich, MA, USA).

PCR products were purified with the Qiagen Gel Extraction Kit (Qiagen, Hilden, Germany). Sequencing libraries were generated using the NEBNext Ultra DNA Library Prep^®^ Kit and Illumina index codes. Library quality was assessed on the Qubit@ 2.0 Fluorometer (Thermo Scientific, Wilmington, DE, USA) and Agilent Bioanalyzer 2100 system (Agilent Technologies, Santa Clara, CA, USA). Libraries were pooled in equimolar concentrations and then sequenced in a paired-end (2 × 250 bp) sequencing format with a MiSeq Reagent Kit V2 using the Novaseq 6000 platform (Illumina, San Diego, CA, USA). Sequencing was performed by Novogene Corporation, Inc. (Sacramento, CA, USA).

### 2.3. Data Analysis

Raw sequences were processed with the DADA2 R package, which was used to resolve amplicon sequence variants (ASV; [38]). For 16S sequences, the following filtering criteria were included: (i) an error threshold of one base and two bases in sense and antisense reads, respectively, and (ii) the removal of sequences with ambiguous bases. Error modeling was performed using the filtered sequences [38]. The matched sequences were merged and filtered to remove chimeric sequences using the “removeBimeraDenovo” function with the “consensus” method [38]. The resulting sequences were used to obtain the consensus sequences. Taxonomic assignment was then performed with the Bayesian classification method [39] using the SILVA database version 138 [40].

The raw ITS reads were also filtered with the DADA2 R package. For this, sequences with ambiguous bases for any of the read pairs were first removed, allowing only 2 errors per read, and adapters were removed with the cutadapt program [41]. After noise filtering, the sequences were matched, and chimeric sequences were removed using the method described above. The sequences thus processed were taxonomically assigned employing the Bayesian classification method [38] against the UNITE ITS databases [42]. The results of both cases (16S and ITS) were integrated into their respective metadata in phyloseq objects with the phyloseq package [43] using R Studio software version 4.1.1. With the Phyloseq base functions, sequences not identified at the phylum level and those identified as “Mitochondrion” and “Chloroplast” at any taxonomic level were removed [38]. The phyloseq object was transformed into an MPSE object with the MicrobiotaProcess R package [44], and all samples were rarefied using the mp_cal_rarecurve function.

We used the microbiome R package [45] to estimate alpha diversity (Observed, Simpson, and Shannon) in each group of samples according to their origin (i.e., soil, seedling, and adult). To compare alpha diversity between developmental stages, we used a Wilcoxon test with a *p*-value cutoff of 0.05. Beta diversity was assessed using a PERMANOVA (999 permutations) based on Bray distances. An estimated PCoA was performed with Hellinger abundances and Bray distances to visualize the clustering pattern of the data according to origin. A table of relative abundances was obtained with the transform_sample_counts function of the Phyloseq package, which was applied to each phyloseq object (16S or ITS). To detect enriched genera among conditions, we used a Linear Discriminant Analysis with Effect Size (LEfSe v 1.0), which was performed with the microbiomeMarker R package [46]. A *p*-value cutoff of 0.05 was used for both the Wilcoxon and Kruskal–Wallis tests. In all cases, plots were customized with the functions of the ggplot2 package v3.5 [47].

## 3. Results

**Figure 1:**
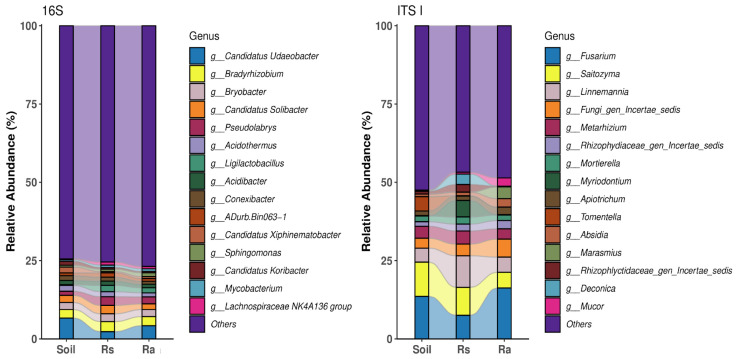
Relative abundances of bacteria and fungi in soil, seedling rhizosphere, and adult rhizosphere samples. The top 15 most dominant fungal and bacterial genera are shown for each ecological compartment analyzed for both 16S and ITS markers. Soil = Bulk soil sample. Rs = seedling rhizosphere sample. Ra = adult rhizosphere sample.

**Figure A1:**
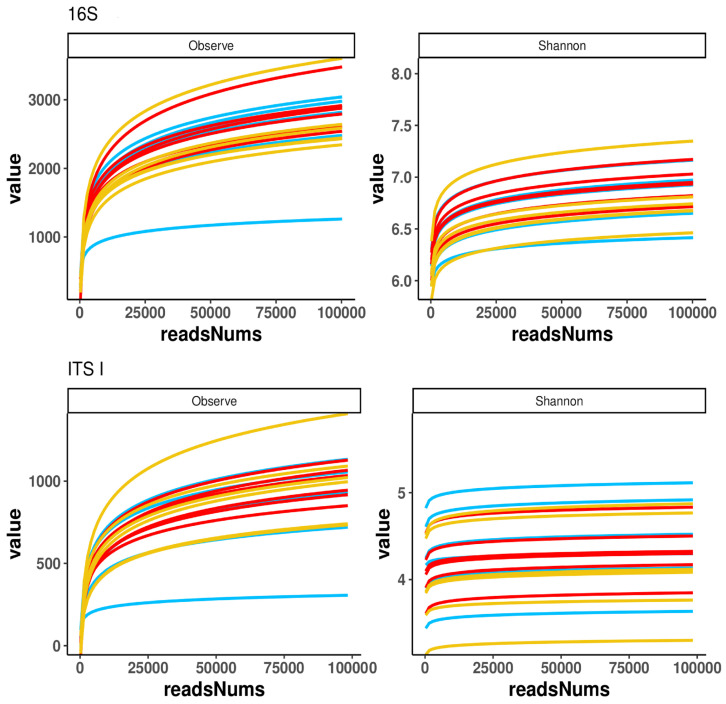
Rarefaction curves of the sequencing libraries. All samples reached the plateau phase.

We compared the assemblage of bacterial and fungal communities associated with the soil and rhizosphere of *C. odorata* at two developmental stages. The characterization included an evaluation of the physicochemical characteristics of the soil, which are presented in Table A1. The raw data obtained in the sequencing experiment were uploaded to the NCBI under the PRJNA1209972 BioProject. Given that the rarefaction curve reached the plateau phase, we considered the sequencing effort to be sufficient for all samples (Figure A1). Based on the relative abundances of the 15 most abundant bacterial/fungal taxa, we determined that the dominant bacterial genera shared among the three compartments (i.e., soil, seedling rhizosphere, and adult rhizosphere) were *Candidatus* Udaeobacter, *Bradyrhizobium*, *Bryobacter*, and *Candidatus* Solibacter, while the most dominant fungal genera were *Fusarium*, *Saitozyma*, and *Linnemania* (Figure 1).

**Figure 2:**
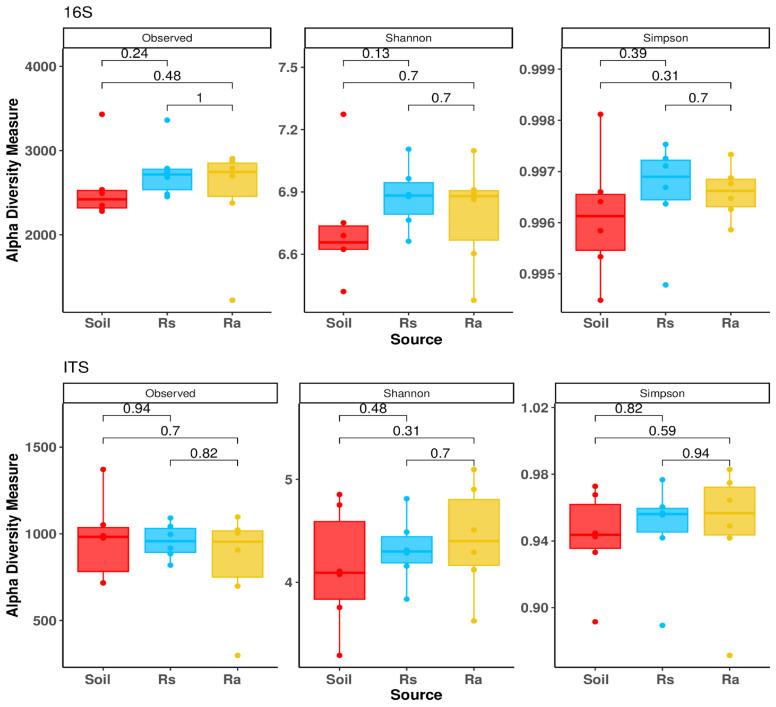
Observed diversity, Shannon’s index, and Simpson’s index obtained from the 16S and ITS gene amplicon analysis. Soil (Soil), seedling rhizosphere (Rs), and adult rhizosphere (Ra) samples.

**Figure 3:**
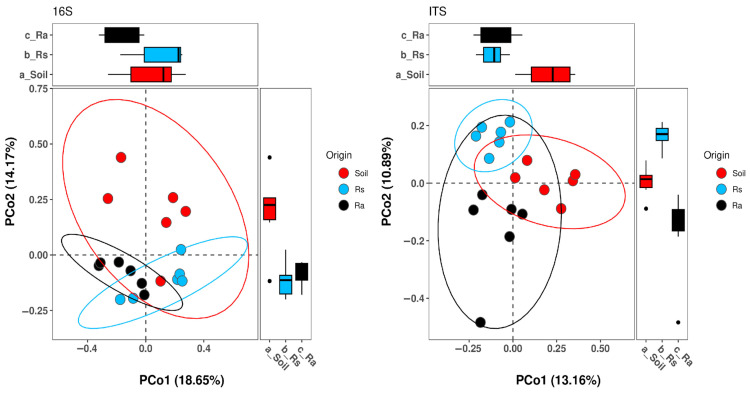
Principal coordinates analysis of microbial communities using 16S and ITS gene amplicons. Soil (Soil), seedling rhizosphere (Rs), and adult rhizosphere (Ra).

Unlike alpha diversity, where no significant differences were found in any of the comparisons (Figure 2), beta diversity showed significant changes (Figure 3; Table 1), since both bacterial and fungal communities were grouped according to the origin of the samples. This clustering pattern is consistent with the results of the PERMANOVA analysis, which showed significant differences (*p* < 0.05) in all comparisons, indicating differences in the structure of the microbial community in each compartment analyzed for both bacterial and fungal taxa.

**Figure 4:**
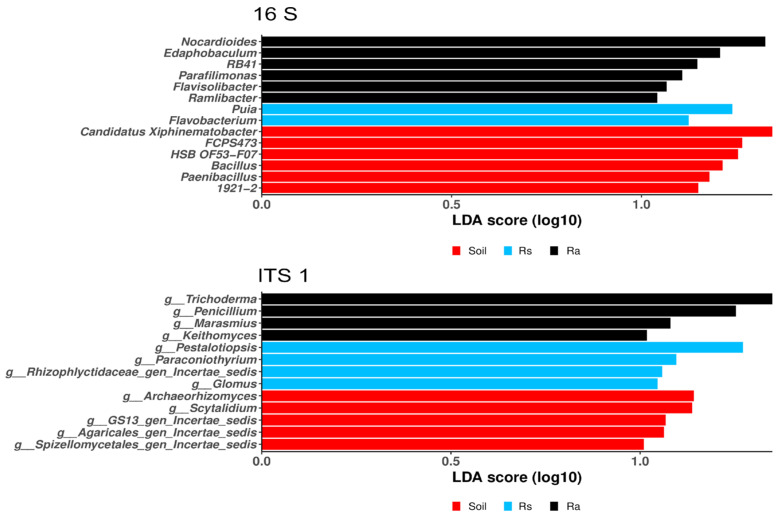
Linear discriminant analysis with effect size (LEfSe) of the 16S and ITS barcodes. Soil (Soil), seedling rhizosphere (Rs), and adult rhizosphere (Ra).

According to the differential analysis, there were significant differences in the relative abundance of bacteria and fungi among the three compartments analyzed (Figure 4). In each case, the finding that differential taxa differed between seedling and adult plants is particularly relevant, as it suggests a reconfiguration in the recruitment of microorganisms according to the developmental stage of the plants. In the case of bacteria, we detected a higher abundance of the genera *Puia* and *Flavobacterium* in seedlings and the genera *Nocardioides*, *Edaphobaculum*, *Parafilimonas*, *Flavisolibacter*, and *Ramlibacter* in adults. In the case of fungi, we observed a similar pattern, with a higher abundance of the genera *Trichoderma*, Penicillium, Marasmius, and Keithomyces in adults and the genera *Pestalotiopsis*, *Paraconiothyrium*, *Rhizophlyctidaceae gen Incertae sedis*, and *Glomus* in seedlings.

## 4. Discussion

The rhizosphere microbiota can serve numerous functions for plants, and its structure is highly dependent on multiple factors, including host plant age. In the present study, we surveyed the rhizosphere microbiota of *C. odorata*, and our findings suggest that this species generates pressure on the bacterial communities surrounding its roots, likely favoring the recruitment of bacteria and fungi that benefit the plant. Moreover, the significant differences found between adults and seedlings indicate that the developmental stage of the Spanish cedar is an important driver of the community assembly process.

For both bacteria and fungi, no significant differences were found in the evaluated parameters of alpha diversity (observed diversity, Shannon index, and Simpson index). Seedlings are small and could exert a lesser influence on soil microbiota than adults. On the other hand, adults, despite exerting a greater influence on the soil microbiota, did not have significantly different alpha diversity from the soil. This may be due to the open nature of the rhizosphere to soil. However, the fact that a similar diversity was observed, the beta diversity differences imply a selection process that is reflected in the differences in relative abundances.

We identified several microbial taxa that, based on the literature, could be potentially beneficial for the cedar. Two examples of this are the genera *Bradyrhizobium* and *Pseudolabrys*, which are nitrogen-fixing bacteria that can be found both free-living in the soil, and associated with plants [48,49]. However, the potentially beneficial effect of these and other microbial taxa must be experimentally further proven.

We detected many acidophilus bacteria, such as *Acidothermus*, *Acidibacter*, and *Ligilactobacillus*, which is consistent with the acidic pH determined for the soil [50,51]. This is not unexpected, as soil pH is the primary shaper of soil microbiota [52]. For example, *Candidatus* udaeobacter has been reported in very acidic soils [53,54], as well as the acid-tolerant genus *Bryobacter* [55]. These species, along with *Acidothermus* and *Candidatus* solibacter, serve as bioindicators of soil acidity [56,57]. Even though these genera are not exclusively found in the rhizosphere of plants, they are part of the soil and it is in line with the fact that the Spanish cedar prefers to grow on slightly acidic soils [58,59].

We found differential genera that were differentially enriched in seedlings and adults. We identified *Puia* and *Flavobacterium* in seedlings; the former has been reported in acid forest soils [52,60,61], but its function in the soil remains unknown. The genus *Flavobacterium* includes species able to degrade complex organic compounds and others that are antagonists of many plant pathogens [62]. In the case of adult plants, we identified bacteria belonging to the genus *Nocardioides*, which have been reported to be growth-promoting bacteria [63,64]. Interestingly, *Parafilimonas*, *Puia*, and *Flavisolibacter* belong to the family Chitinophagaceae, which contains several members that have been previously identified in root microbiomes and may be root beneficiaries [65,66,67] and protect plants against fungal pathogens, since they harbor enzymes such as chitinases that are involved in fungal cell wall degradation [68]. Members of this family may also decompose cellulose and benefit from root turnover [69]. These bacteria may thus be beneficial for the plant, likely acting as pathogen suppressors, but again, to assess their beneficial properties, future studies should test the inocula of these taxa on seedlings of *C. odorata*.

In the case of the fungal community, we observed a similar pattern, as the assembly was found to be influenced by the development of the plants. Adult plants showed a significantly higher abundance of *Trichoderma* spp., which is a genus widely recognized as a potent suppressor of pathogenic microorganisms and a promoter of plant growth [70]. This genus uses various direct and indirect biocontrol mechanisms against both biotic and abiotic stresses [71]. This could also be the case for the enrichment of *Penicillium*, which is one of the most common genera of rhizosphere fungi and performs a wide range of functions that benefit many plant species, since it produces solubilized phosphorus, siderophores, and phytohormones such as indole acetic acid and gibberellic acid [72]. Pathogenic genera such as *Fusarium*, which includes many species that cause disease and damage to plants and seeds [73] were found as dominant taxa in all the analyzed compartments. This result is not surprising since *Fusarium* genus is a ubiquitous fungus widely distributed in the environment and frequently inhabits the soil for a long time in the form of chlamydospores. Because of this, it can be present in the rhizosphere and bulk soil, although not necessarily causing infection in the plant, since all the sampled specimens were healthy.

The enrichment of potentially beneficial genera in the rhizosphere of adults may be advantageous, but the increased abundance of some genera may also reflect changes in the microenvironment, as is the case of *Marasmius*, whose enrichment in the rhizosphere of adults is likely due to its saprophytic lifestyle, which involves the degradation of organic matter [74,75]. The enrichment of the genus *Keithomyces* in adult plants is of particular interest, as some members of this genus have entomopathogenic effects against lepidopteran larvae [76] and therefore warrant further investigation to determine whether their presence helps adult plants resist insect attacks.

In contrast to adults, the seedling rhizosphere not only contained genera with antagonistic effects but also exhibited an enrichment of species with pathogenic potential, such as those of the genus *Pestalotiopsis*, which are important causal agents of plant diseases [77]. Depending on their respective habitat, they can have different ecological functions. As endophytes in plants or saprophytic fungi, they can cause post-harvest diseases [78], and they are very common in forest nurseries where their hosts are cultivated, causing necrotic leaf lesions that, although they do not always result in death, can delay seedling growth, and some species can cause regressive death [79]. Thus, evaluating the pathogenic effects of *Pestalotiopsis* species on Spanish cedar seedlings would be useful. In contrast, *Glomus*, a genus of arbuscular mycorrhizal fungi, may be beneficial in the early stages of Spanish cedar development, and its effect as a plant growth promoter [80,81] merits further study. However, it is interesting to note that, in general, the rhizosphere of *C. odorata* is an important reservoir of Glomeromycota fungi, hosting more than 50 taxa [33,34,35], which represents, for example, 83% of the total number of Glomeromycota species reported in Costa Rica [35] or 31% of the species known in Mexico [82]. Nevertheless, Glomeromycota fungi are generally poorly represented in the microbial communities found in the rhizosphere of *C. odorata* adults and seedlings.

In light of these findings, it is probable to hypothesize that Spanish cedar adults recruit a specific microbiota that includes fungal and bacterial taxa with pathogen-suppressive capacities that help them combat microbial pathogens, most likely fungal in nature. In contrast, seedlings do not have this capacity, likely due to an ongoing ecological succession process that leads to the onset of a stable microbiota that is consolidated in the adult stage. In both cases, the recruitment and filtering processes could begin early in the development of the Spanish cedar rhizosphere and continue into adulthood, which leads to differentiation from the surrounding bulk soil microbiota. However, we encourage experimental testing of these ideas, for example, by doing microbial transplant experiments from adult plants to seedlings and testing if adult rhizospheric soils have suppressive potential to avoid the establishment of phytopathogens in the early stage of cedar plants. Lastly, our study has at least two limitations: we do not offer information to elucidate the variables that account for the observed variation in changes to the microbiota. Experiments must be conducted to elucidate the alterations in the plant’s communication processes with the soil as influenced by age and to correlate variations in plant age with modifications in soil structure and properties. In this sense, extending the analysis as we have done in this work at a biogeographical scale involving the analysis of a higher number of samples and replicate could be advantageous.

## 5. Conclusions

We found that *Cedrela odorata* exerts strong pressure on the assembly of its rhizosphere microbiota, which likely follows a development-dependent process, as the structure of the microbiota clearly differed among compartments and between the compared developmental stages. The differences found in terms of the most abundant genera in each compartment suggest that the enriched taxa perform specialized functions according to each developmental stage. However, it appears that the process that leads to the establishment of the potentially pathogen-suppressive microbiota observed in adult plants is not immediate but requires a gradual recruitment of beneficial microorganisms throughout development.

We believe that the information obtained in the present study will facilitate the selection of potentially beneficial bacterial and fungal genera for the formulation of microbial consortia aimed at improving the development of *C. odorata* seedlings for reforestation purposes.

## Figures and Tables

**Table 1:** PERMANOVA analysis based on Bray–Curtis distances to compare the beta diversity among comparison groups.

16S						
	d.f.	SumOfSqs	R^2^	F	Pr(>F)	
Origin	2	0.837	0.218	2.09	0	
Residual	15	3.001	0.782			
Total	17	3.838	1.000			
Groups		measure	F	R^2^	*p*-value	*p*-adjusted
Adult rhizosphere vs. Seedling rhizosphere		Bray	2.140	0.176	0.02	0.02
Adult rhizosphere vs. Soil		Bray	1.865	0.157	0.01	0.02
Seedling rhizosphere vs. Soil		Bray	2.314	0.188	0	0.01
ITS						
	d.f.	SumOfSqs	R^2^	F	Pr(>F)	
Origin	2	0.898	0.194	1.809	0	
Residual	15	3.723	0.806			
Total	17	4.620	1.000			
Groups		measure	F	R^2^	*p*-value	*p*-adjusted
Adult rhizosphere vs. Seedling rhizosphere		Bray	1.940	0.162	0	0.01
Adult rhizosphere vs. Soil		Bray	1.559	0.135	0.01	0.01
Seedling rhizosphere vs. Soil		Bray	1.963	0.164	0	0.01

## Data Availability

The original contributions presented in this study are included in the article. Further inquiries can be directed to the corresponding authors.

## Appendix A

**Table A1:** Physicochemical characteristics of the soil at the study site.

Quantified Element	Value
% Sand	31.32
% Clay	28.72
% Silt	39.96
Saturation point	57.20%
Field capacity	42.90%
Permanent wilting point	22.50%
Apparent density	1.03 g/cm^3^
pH	4.97
M.O.	3.97%
K	240.46 ppm
Ca	1377.54 ppm
Mg	245.30 ppm
Na+	17.96 ppm
Fe	47.53 ppm
Zn	1.00 ppm
Mn	4.32 ppm
Cu	0.42 ppm
P-Olsen	8.16 ppm
Total Nitrogen	0.25%
